# Comparative effectiveness of cognitive behavioral therapy for insomnia: a systematic review

**DOI:** 10.1186/1471-2296-13-40

**Published:** 2012-05-25

**Authors:** Matthew D Mitchell, Philip Gehrman, Michael Perlis, Craig A Umscheid

**Affiliations:** 1 Center for Evidence-based Practice, University of Pennsylvania Health System, 3535 Market St. Mezzanine Suite 50, Philadelphia, PA 19104, USA; 2 Behavioral Sleep Medicine Program, Department of Psychiatry, University of Pennsylvania Perelman School of Medicine, 3535 Market St., Suite 670, Philadelphia, PA 19104, USA; 3 Center for Sleep and Circadian Neurobiology, University of Pennsylvania Perelman School of Medicine, 3624 Market St., Philadelphia, PA 19104, USA; 4 Center for Clinical Epidemiology and Biostatistics, University of Pennsylvania Perelman School of Medicine, Blockley Hall, 423 Guardian Dr., Philadelphia, PA 19104, USA; 5 Division of General Internal Medicine, Department of Medicine, University of Pennsylvania Perelman School of Medicine, 3400 Spruce St., Philadelphia, PA 19104, USA; 6 Leonard Davis Institute of Health Economics, University of Pennsylvania, 3641 Locust Walk, Philadelphia, PA 19104, USA

**Keywords:** Insomnia, Sleep, Behavior therapy, Cognitive therapy, Hypnotics and sedatives

## Abstract

**Background:**

Insomnia is common in primary care, can persist after co-morbid conditions are treated, and may require long-term medication treatment. A potential alternative to medications is cognitive behavioral therapy for insomnia (CBT-I).

**Methods:**

In accordance with PRISMA guidelines, we systematically reviewed MEDLINE, EMBASE, the Cochrane Central Register, and PsycINFO for randomized controlled trials (RCTs) comparing CBT-I to any prescription or non-prescription medication in patients with primary or comorbid insomnia. Trials had to report quantitative sleep outcomes (e.g. sleep latency) in order to be included in the analysis. Extracted results included quantitative sleep outcomes, as well as psychological outcomes and adverse effects when available. Evidence base quality was assessed using GRADE.

**Results:**

Five studies met criteria for analysis. Low to moderate grade evidence suggests CBT-I has superior effectiveness to benzodiazepine and non-benzodiazepine drugs in the long term, while very low grade evidence suggests benzodiazepines are more effective in the short term. Very low grade evidence supports use of CBT-I to improve psychological outcomes.

**Conclusions:**

CBT-I is effective for treating insomnia when compared with medications, and its effects may be more durable than medications. Primary care providers should consider CBT-I as a first-line treatment option for insomnia.

## Background

The prevalence of insomnia in primary care patients is as high as 69% [1,2] compared to 33% in the general population [3]. Insomnia can exist as a primary disorder or co-morbid with other conditions including depression [4] and chronic pain. In the past insomnia was considered to be a symptom of these conditions with the assumption that treatment of these ‘primary’ conditions would lead to the resolution of insomnia, eliminating the need for targeted insomnia treatment. There is now evidence to suggest that insomnia often persists following resolution of these ‘primary’ conditions, and that it generally does not spontaneously resolve over time if left untreated [5]. Insomnia is independently associated with significant morbidity including fatigue, impaired concentration and memory, irritability, difficulty in interpersonal relationships, decreased quality of life, and increased risk of new-onset psychiatric illness [1,6-9]. In addition, there is evidence that insomnia may confer risk for medical illness including hypertension, heart disease, and diabetes [10,11], and is associated with increased overall health care costs [9].

The most common approach to the management of insomnia is medication treatment. Numerous trials have documented moderate efficacy with benzodiazepine receptor agonists [12,13]. The advantages of medications are that they are widely available and, when effective, lead to clinical improvement rapidly. The disadvantages are the potential for side-effects, dependence, and tolerance over time. Perhaps the most important disadvantage is that medications are usually not curative, leading to long-term treatment over many years despite a lack of safety and efficacy data for their long-term use beyond 1–2 years.

An alternative treatment approach is cognitive behavioral therapy for insomnia (CBT-I). CBT-I is a non-pharmacological approach to treatment comprised of several strategies. The goal of CBT-I is to target those factors that may maintain insomnia over time, such as dysregulation of sleep drive, sleep-related anxiety, and sleep-interfering behaviors. This is accomplished by establishing a learned association between the bed and sleeping through stimulus control, restoring homeostatic regulation of sleep through sleep restriction, and altering anxious sleep-related thoughts through cognitive restructuring. By changing sleep-related behaviors and thoughts, CBT-I may target those factors that cause insomnia to persist over time. CBT-I is delivered over the course of 4–8 sessions that occur weekly or every other week for 30–60 minutes each. There are two main disadvantages to CBT-I. First, during the first few weeks of treatment there is often an acute reduction in total sleep time that can lead to the side effect of increased daytime sleepiness which, for some, is enough to lead them to drop out of treatment. Second, improvements from CBT-I are typically not seen until 3–4 weeks into treatment. While a few research studies have examined the efficacy of nurse-led CBT-I in primary care settings [14], in current clinical practice it is usually necessary to refer out to individuals with specialized training in this treatment. It should be noted that the core therapies for CBT-I substantially differ from other forms of CBT and it is for this reason that the abbreviation CBT-I denotes this form of CBT specifically for insomnia [15].

Treating insomnia with CBT-I, as opposed to medication, has a number of potential advantages, including fewer known side effects, and an explicit focus on treating the factors that may be responsible for perpetuating chronic insomnia in an effort to produce more durable effects. Some patients also prefer non-medication treatments [12,16,17]. Providers often have negative attitudes towards hypnotics as well, and prefer to reduce their prescriptions of such medications [18]. These advantages of CBT-I suggest that it might be a viable option for the treatment of insomnia [12,17].

Evidence suggests that CBT-I is effective when compared with placebos [19,20]. A new systematic review [21] meta-analyzes evidence from 14 randomized trials comparing groups of patients given CBT-I to control groups. A variety of controls were combined in that analysis, such as patients on a waiting list to receive CBT-I and patients offered a sleep hygiene program which has been shown to be of little or no benefit [17]. The meta-analysis found that CBT-I had a medium to large effect in reducing insomnia, and that the effects were maintained even after conclusion of the treatment period. But this review does not tell us whether CBT-I is as effective as sleep medications. The AHRQ Evidence-based Practice review [20] addressed that question, but it was published in 2005 and lacks evidence published since September 2004.

The primary objective of this review is to examine the most up-to-date evidence comparing CBT-I to medications in patients with primary and comorbid insomnia and assess the comparative effectiveness of these treatments.

## Methods

### Inclusion criteria

We considered only published randomized controlled trials (RCTs) with no exclusions based on date of publication or language. Inclusion criteria were defined in advance of data abstraction using the PICO framework below [22].

#### Patients

Included studies examined patients aged 18 or older diagnosed with chronic insomnia per DSM-IV criteria. Studies of patients with medical or psychological comorbidities were included but analyzed separately, as were studies of patients already using medications for insomnia. Studies with fewer than 10 patients in each group were excluded.

#### Intervention

Study patients had to be treated with either individual or group CBT-I to be included. Therapy had to be delivered by a professional: studies of self-help programs were excluded. Studies with telephone or internet sessions were included if those sessions were supplementary to in-person CBT-I. We did not set minimum or maximum treatment periods for study inclusion. Studies that were designed to compare individual elements of a CBT-I program to each other were excluded.

#### Comparison

We examined all RCTs comparing CBT-I to FDA approved prescription or non-prescription medications used to treat insomnia both on- and off- label (e.g. benzodiazepines, non-benzodiazepine receptor agonists, antidepressants, and antihistamines).

#### Outcomes

Studies had to report at least one quantitative measure of sleep to be included in this analysis. These measures included sleep latency, wake after sleep onset, sleep efficiency, total sleep time, and total wake time. Measures from patients’ sleep diaries and measures from automated means (polysomnography and actigraphy) were analyzed separately because of known unreliability in patients’ perceptions of sleep. As secondary outcomes, standardized measures of quality of life, sleep quality, and psychological outcomes including depression, anxiety, and fatigue were also abstracted when available, as was data on adverse events.

### Literature searches and study inclusion/exclusion

We searched Medline (OVID interface, Additional file 1: Table S1), the Cochrane Central Register ( Additional file 1: Table S2), EMBASE (Additional file 1: Table S3), and PsycINFO (Additional file 1: Table S4). Search strategies combined indexing terms for CBT with indexing terms for insomnia, then applied filters for randomized trials. We did not limit the search to specific drugs. Detailed syntax of each search is reported in the supplement. All searches were completed in September 2011. We also reviewed published systematic reviews [19,20,23] for eligible RCTs which may have been missed by our searches: none were found.

**Figure 1:**
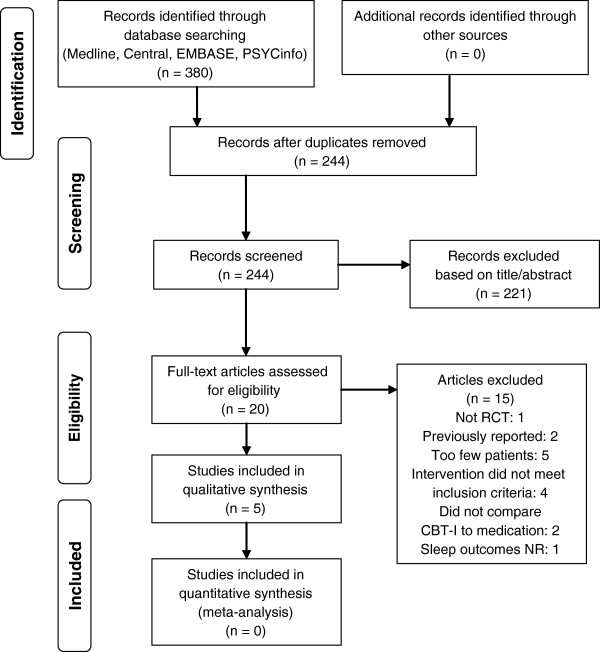
PRISMA flow diagram for literature search and article inclusion.

Disposition of the search hits and articles retrieved is shown in Figure 1. Two research analysts (MDM, BL) reviewed full text of each retrieved article to determine whether it met the stated inclusion criteria. Disagreements were resolved by a third reviewer (PG). Reasons for exclusions of studies are summarized in Figure 1 and the complete list of excluded articles with reasons for exclusion are in Additional file 1: Table S5.

### Data abstraction and analysis

Study characteristics were abstracted into a database with fields for study setting, patient selection criteria, CBT-I interventions, and reviewers’ comments. This database helped us decide how to organize studies and evidence tables. Study results were then abstracted directly into evidence tables. Data synthesis methods followed the practice of the Cochrane Collaboration. Assessment of RCT quality was done using a nine-point scale (Additional file 1: Table S6) based on scales by Jadad [24] and Chalmers [25]. Quality ratings for each individual study are shown in Additional file 1: Table S7. The overall quality of the evidence base for each comparison was rated using the GRADE approach [26-28].

### Outcome measures

Key quantitative measures of sleep are sleep latency (the amount of time it takes for a person to fall asleep after retiring to bed), total sleep time (TST), time spent awake after sleep onset, total wake time during the sleep period, and sleep efficiency (the percentage of the sleep period during which the patient is actually asleep). It should be noted that TST is not only an outcome variable, but is manipulated as part of CBT-I via the restriction of time spent in bed. Specifically, sleep restriction therapy requires that time in bed be reduced to a time interval that equals the patient’s ‘sleep ability’ by measuring average TST during a two-week baseline period. The net result of this, after completion of CBT-I, is that many patients do not recover their baseline TST but are nevertheless substantially improved with respect to other aspects of sleep. This said, it is important to assess TST as an outcome measure so that the effects of CBT-I are fully characterized. There can also be substantial differences between automated measures of sleep time and patients’ self-reports on sleep diaries.

Most studies reported these sleep outcomes for multiple time points. Where available, we tabulated results for two of those points from each study: the time period immediately following treatment, which for CBT-I was typically 4 to 8 weeks from the start of therapy, and for the longest follow-up period reported by study authors, which was typically six to twelve months.

As secondary outcomes, we also analyzed results from standardized patient questionnaires on sleep and insomnia symptoms, such as the Pittsburgh Sleep Quality Index. Non-standard instruments, such as asking the patient to grade his or her sleep quality on a scale of 1 to 5, were not analyzed.

Daytime outcomes analyzed included standardized and validated measures of patients’ general quality of life, and measures of psychological outcomes including depression, anxiety, and fatigue.

## Results

Our search found a total of five RCTs that met the inclusion criteria and compared CBT-I to medications. The five trials all involved patients with primary insomnia. The trials compared CBT-I to zopiclone [29], zolpidem [30], temazepam [31,32], and triazolam [33]. The design and quality of these studies is reported in Table 1 and their short and long term results are summarized in Table 2 and Table 3 respectively. Only one trial compared CBT-I to drug therapy in patients with comorbid insomnia [34]: the drug used was nefazodone (now withdrawn from the market), so the study was excluded from further analysis. The evidence base was too small and heterogeneous for meta-analysis.

**Table 1:** Studies comparing CBT-I to pharmacological therapies: methods

**Study**	**Design**	**Patients**	**Intervention and duration**	**Comparison**	**Sleep measurements reported**	**Comment**
**Location**	**Quality**	**Longest follow-up**				
**CBT-I vs. zopiclone**						
Sievertsen 2006 [29]	RCT	46 patients, age 55 and up	Individual CBT-I,6 weekly sessions	Zopiclone, 7.5 mg nightly	Sleep diaries, polysomnography	Study also included placebo group. Zopiclone patients had option to continue it after 6 week period.
Norway	5	12 months				
						Daytime outcomes reported in [35]
**CBT-I vs. zolpidem**						
Jacobs 2004 [30]	RCT	63 patients, age 25-64	Individual CBT-I, 5 sessions, 6 weeks; plus 1 telephone session	Zolpidem, see comment	Sleep diaries, sleep monitor	Dose 10 mg→5 mg→5 mg q2d over 6 week period.
USA	5	12 months				
**CBT-I vs. temazepam**						
Wu 2006 [32]	RCT	77 patients	Individual CBT-I 2 per week, 8 weeks	Temazepam, see comment	Sleep diaries, polysomnography	Dose 7.5 mg→30 mg→15 mg over 8 week period
China	3	8 months				
						Study also included placebo and combined therapy groups
Morin 1999 [31]	RCT	78 patients, age 55 and up	Group CBT-I 8 weekly sessions	Temazepam, see comment	Sleep diaries, polysomnography	Dose 7.5 mg→30 mg as needed over 8 week period
						Study also included placebo and combined therapy groups.
Canada	6	24 months				
						Adverse effects reported in [36] Patient attitudes reported in [37]
**CBT-I vs. triazolam**						
McCluskey 1991 [33]	RCT	30 patients	Group CBT-I 2 per week, 3 weeks	Triazolam, 0.5 mg, then tapered to 0	Sleep diaries	Triazolam group also had weeklygroup meetings but no CBT-I
USA	4	9 weeks				

**Table 2:** Studies comparing CBT-I to pharmacological therapies: post-treatment results

**Study** **Follow-up & method**	**Group, N**	**Sleep latency**	**Total sleep time**	**Total wake time**	**Sleep efficiency**	**Other**	**Adverse effects**	**Notes**
**CBT-I vs. zopiclone**						SWS		
Sievertsen 2006 [29] 6 weeks	CBT-I: 18	Not reported	–26.2 min	–56.4 min	+7.5%	+17.2 min	None	
	Zopiclone: 16		–65.6 min	–3.9 min	–0.8%	–15.1 min	1 withdrawal	
			p = NS	p < 0.001	p = NS	p = 0.002		
Polysomnography								
Sleep diary	CBT-I: 18	Not reported	+16.9 min	–48.3 min	+11.8%			
	Zopiclone: 16		+34.6 min	–25.8 min	+8.1%			
			p = NS	p = NS	p = NS			
**CBT-I vs. zolpidem**								
Jacobs 2004 [30]	CBT-I: 13	–15.5 min	–2.6 min	Not reported	+5.5%		No withdrawals due to side effects	p values based on number of patients with satisfactory latency or efficiency
8 weeks	Zolpidem: 12	–6.1 min	–51.6 min		+2.1%			
Sleep monitor		p = NS	p = NR		p = NS			
Sleep diary	CBT-I: 13	–33.8 min	+48.6 min	Not reported	+17.3%			
	Zolpidem: 12	–12.8 min	+69.2 min		+2.1%			
		p < 0.05	p = NR		p = 0.007			
**CBT-I vs. temazepam**						WASO		
Wu 2006 [32]	CBT-I: 19	–35.9 min	+21.6 min	Not reported	+9.2%		Not reported	p values based on post-intervention differences
8 weeks	Temazepam: 17	–44.9 min	+66.5 min		+14.3%			
Polysomnography		p < 0.01	p < 0.004		p < 0.05			
Sleep diary	CBT-I: 19	–37.0 min	+38.7 min	Not reported	+13.4%			
	Temazepam: 17	–53.2 min	+73.5 min		+15.1%			
		p < 0.001	p < 0.01		p < 0.01			
Morin 1999 [31]	CBT-I: 18	Not reported	+6.8 min	Not reported	+8.5%	–32.5 min	Not reported	
8 weeks	Temazepam: 17		+35.3 min		+7.5%	–23.3 min		
Polysomnography			p = NS		p = NS	p = NS		
Sleep diary	CBT-I: 18	Not reported	+30.5 min	Not reported	+16.5%	–27.3 min		
	Temazepam: 17		+43.7 min		+10.3%	–25.6 min		
			p = NS		p = NS	p = NS		
**CBT-I vs. triazolam**								
McCluskey 1991 [33]	CBT-I: 15	–44 min	+40 min	Not reported	Not reported		Not reported	
3 weeks	Triazolam :15	–45 min	+57 min					
Sleep diary		p = NS	p = NS					

*SWS* Slow wave sleep, *WASO* Wake after sleep onset.

**Table 3:** Studies comparing CBT-I to pharmacological therapies: follow-up results

**Study**	**Group**	**Sleep latency**	**Total sleep time**	**Total wake time**	**Efficiency**	**Other**	**Notes**
**CBT-I vs. zopiclone**						SWS	
Sievertsen 2006 [29]	CBT-I: 18	Not reported	–5.0 min	–60.7 min	+8.7%	+21.1 min	
	Zopiclone :16		–56.2 min	–9.9 min	–0.4%	–17.6 min	
6 months			p = NS	p = 0.01	p = 0.008	p = 0.001	
Polysomnography							
Sleep diary	CBT-I: 18	Not reported	+42.4 min	–73.3 min	+14.2%		
	Zopiclone :16		+40.5 min	–42.2 min	+10.7%		
			p = NS	p = 0.03	p = NS		
**CBT-I vs. zolpidem**							
Jacobs 2004 [30]	CBT-I: 8						No long-term follow-up for zolpidem group
12 months	Zolpidem: none						
Sleep diary							
**CBT-I vs. temazepam**						WASO	
Wu 2006 [31]	CBT-I: 19	–32.8 min	+30.3 min	Not reported	+10.2%		p values based on post-intervention differences
8 months	Temazepam: 17	–17.2 min	–13.0 min		–1.9%		
Polysomnography		p < 0.004	p < 0.05		p < 0.01		
Sleep diary	CBT-I: 19	–41.8 min	45.5 min	Not reported	+16.8%		
	Temazepam: 17	–20.5 min	–6.0 min		+3.9%		
		p < 0.003	p < 0.01		p < 0.05		
Morin 1999 [31]	CBT-I: 13	Not reported	+65.2 min	Not reported	+16.4%	–16.5 min	All measurements in temazepam group significantly worsened from end of treatment to end of follow-up.
24 months							
Sleep diary	Temazepam: 12		+11.5 min		+2.9%	–4.6 min	
			p = NR		p = NR	p = NR	
**CBT-I vs. triazolam**							
McCluskey 1991 [33]	CBT-I: 15	–45 min	+51 min	Not reported	Not reported		
8 weeks	Triazolam :15	–21 min	+14 min				
Sleep diary		p < 0.01	p = NR				

*SWS* Slow wave sleep, *WASO* Wake after sleep onset.

### Quantitative sleep outcomes

All trials asked patients to complete sleep diaries. All but one also used polysomnography or actigraphs to objectively measure sleep outcomes. Reported sleep measures included sleep latency, TST, and total time spent awake during the night. Our results tables show the mean changes in these variables from baseline to the end of the active treatment period (Table 2) and the end of the study follow-up period. Some studies reported intermediate time points as well, which were consistent with the initial and final results.

The evidence comparing benzodiazepines to CBT-I in the short term is of very low grade (Table 4). One study (meeting 3 of 9 quality points) found the benzodiazepine temazepam to be statistically more effective than CBT-I for improving sleep latency and sleep efficiency, while another (meeting 6 of 9 quality points) found no difference (Table 2). Conversely, there is moderate grade evidence suggesting CBT-I is superior to the non-benzodiazepines zopiclone and zolpidem for improving sleep measures in the short term.

**Table 4:** Evidence summary and GRADE analysis

**Comparison**	**Outcome period (quantitative sleep measures)**	**Conclusion**	**Quantity and type of evidence**	**Starting level of evidence strength**	**Quality**	**Inconsistency**	**Directness**	**Sparse or imprecise**	**Reporting bias**	**Strong or very strong association**	**Dose-resp.**	**Confounders would increase eff.**	**Final level of evidence strength**
CBT-I vs. benzodiazepines	Short term	Improved less with CBT-I	3 RCT	High	–2	–1	0	0	0	0	0	0	Very low
	Long term	Improved more with CBT-I	3 RCT	High	–1	0	0	0	0	0	0	0	Moderate
CBT-I vs. non-benzodiazepines	Short term	Improved more with CBT-I	2 RCT	High	–1	0	0	0	0	0	0	0	Moderate
	Long term	Improved more with CBT-I	1 RCT	High	–1	0	0	–1	0	0	0	0	Low

Evidence assessed using methods of the GRADE Working Group [26-28].

Evidence strength ratings:

High: Further research is very unlikely to change our confidence in the estimate of effect.

Moderate: Further research is likely to have an important impact on our confidence in the estimate of effect and may change the estimate.

Low: Further research is very likely to have an important impact on our confidence in the estimate of effect and is likely to change the estimate.

Very low: Any estimate of effect is very uncertain.

Short term outcomes typically 4 to 8 weeks, long-term outcomes typically 6 to 12 months.

Long-term studies (6 to 24 months after completion of treatment) consistently favor CBT-I over both benzodiazepines and non-benzodiazepines for improving sleep efficiency, with moderate grade evidence for the former and low grade evidence for the latter. CBT-I generally led to improvements of 30 to 45 minutes in sleep latency and 30 to 60 minutes in total sleep time (Table 3). Sleep efficiency improved 8 to 16 percent with CBT-I, but improved less with drugs. The effects of CBT-I appear to be sustained over time, while the effects of drug therapy decline.

### Patients’ subjective evaluation of sleep

Only two of the studies reported results from standardized sleep questionnaires given to patients at the end of the follow-up period. Both reported that patients were significantly more satisfied with CBT-I than with zopiclone, but they did so using different measures. In Morin’s study [31] the Sleep Impairment Index declined 10 points after 24 months for patients treated with CBT-I, but only 4 points for patients given tamezepam (p = 0.01). Wu et al [32] surveyed patients using the Pittsburgh Sleep Quality Index: after 8 months this measure improved an average of 1.2 points in patients treated with CBT-I, while it worsened 0.8 points in patients given tamezepam (p < 0.01).

### Quality of life and daytime outcomes

Evidence comparing CBT-I and medications for insomnia-related outcomes other than direct sleep measurements is scarce. One trial [35] found no significant difference in overall quality of life (SF-36) between patients treated with CBT-I and patients treated with zopiclone. This study also found no difference in daytime fatigue between the two groups, as measured by patients’ reaction times. Quality of life outcomes were not reported in the other trials.

The same trial also measured patients depression and anxiety symptoms using several standard questionnaires. Six months after treatment, scores were significantly improved on both components of the State-Trait Anxiety Index for CBT-I patients compared to zopiclone patients (state: –5.11 vs. +1.61, p < 0.05; trait: –4.47 vs. +1.97, p < 0.01). Trends towards improved results with CBT-I were observed in the Penn State Worry Questionnaire and the Worry Domains Questionnaire, but the differences between groups were not statistically significant. No difference between groups was observed in the Inventory of Interpersonal Problems.

### Adverse effects of treatment

Reporting of adverse events in all of these trials is limited, so we cannot draw any conclusions regarding the relative safety of CBT-I compared to benzodiazepines and non-benzodiazepines.

We found no trials comparing CBT-I to antihistamines or antidepressants.

## Discussion

Overall, we found that CBT-I is at least as effective for treating insomnia when compared with sleep medications, and its effects may be more durable than medications. The strength of these claims needs to be tempered with a critical evaluation of the available evidence. There have only been a handful of studies that have included both cognitive behavioral and pharmacotherapy treatment arms for inclusion in this systematic review. A significant limitation of the studies is that they all focused on patients with primary insomnia who, while more straightforward for inclusion in clinical studies, may not be representative of the majority of patients who have comorbid medical or psychiatric disorders. RCTs of CBT-I only, with no medication comparator condition, generally find that treatment is as efficacious in those with comorbid disorders compared to those with primary insomnia (for example see [38]). While this suggests that the results of this review may also hold for comorbid insomnia, studies including both CBT-I and medication arms are needed in this patient population. The studies also were diverse in terms of sleep-related outcome measures (subjective versus objective) and demographic composition. In terms of outcome measures, insomnia RCTs generally find larger effects of treatment on subjective measures such as questionnaires than on objective measures of actigraphy and polysomnography. However, this does not limit the meaning of the findings since the diagnosis of insomnia relies on patient report and not on a diagnostic laboratory test. In terms of demographic composition, three of the five studies we included were in older adults. Future studies will need to be conducted in both older and younger adult samples, but it is noteworthy that the findings were generally consistent across studies despite the diversity of subject ages.

The results of this analysis indicate that more research is warranted in this area to better understand the relative efficacy of CBT-I and hypnotics in order to make decisions about best practices.

Even with these limitations, these results have a number of important treatment implications for the primary care setting. First, providers should consider CBT-I as a treatment option for their patients with insomnia. Second, CBT-I may have advantages when compared to medications, most notably more durable treatment gains that reduce or eliminate the need for long-term pharmacologic treatment. In addition, those significant clinical gains can be made in a relatively short number of treatment sessions (most studies were 6–8 weekly or bi-weekly sessions). Future research comparing the efficacy of CBT-I and hypnotics may find that treatment efficacy is moderated by patient characteristics such as age or comorbid diagnoses. These data can lead to the development of treatment algorithms that would allow primary care providers to make more informed treatment decisions. Of note, three of the studies [29,31,32] included a treatment arm that was a combination of CBT-I and medication. Outcomes were generally comparable to those receiving CBT-I alone, but future studies should examine additional strategies for combination treatment that capitalize on the relative advantages of medication (rapid onset on therapeutic response) and CBT-I (long-term durability of treatment gains).

Despite the evidence supporting the effectiveness of CBT-I, many providers may not be aware of its existence or know how to refer patients for treatment [39]. Referral can be accomplished in several ways. First, there is a growing registry of providers who have sought specialized certification in the delivery of CBT-I and other behavioral sleep medicine interventions to whom patients can be referred ( http://www.absm.org/BSMSpecialists.aspx). These providers are primarily clinical psychologists and are located in a variety of settings including sleep disorders centers and private practice [14,40]. Second, primary care practices can partner with local psychologists or others to provide CBT-I. Lastly, a few studies have yielded promising results by implementing CBT-I in the primary care setting by nurses provided the necessary training and supervision [41,42]. By utilizing these various approaches, primary care providers can make CBT-I accessible. Yet, even with these options, getting a patient access to CBT-I may present challenges not encountered when providing a patient with a simple prescription for a sleep medication. First, there can be greater difficulty in getting insurance coverage for CBT-I than for medications. Second, therapists to provide CBT-I may not be available in some parts of the country or the world. However, the evidence reviewed in this manuscript suggests that the additional effort in obtaining access to a therapist if available may be worth the more long-term improvements patients may achieve with CBT-I without the extended use of sleep medications.

The GRADE approach used to grade the evidence base for the comparisons examined in this study can help identify areas where the evidence is least robust and where additional studies can most impact our conclusions about CBT-I. GRADE can also help identify causes for the weakness of the evidence base. Data on long-term outcomes of CBT-I compared to non-benzodiazepines is still sparse, and inconsistencies in the data comparing CBT-I to benzodiazepines in the short term need to be resolved. Data on CBT-I for patients in critical subgroups of interest such as those with comorbid insomnia is sparse as well, and there have been no trials comparing CBT-I to approved drugs in these patients. This is an important limitation of the findings in our review as many patients presenting with insomnia to primary care practices will have secondary or co-morbid insomnia, and thus the findings here may not be as generalizable to those settings. In addition, comparisons of CBT-I to drugs commonly used off-label for the treatment of insomnia, including antidepressants and antihistamines, are not available. Lastly, all studies in this field suffer an unavoidable risk of bias because double-blinding is not possible.

## Conclusion

CBT-I is an effective treatment for insomnia that can produce durable results in a relatively brief number of visits. Low- to moderate quality evidence suggests it has greater effectiveness than medications for treatment of insomnia six months or more after therapy is completed. Additional research is needed to validate its effectiveness in long-term studies beyond 1–2 years and in patients with comorbid insomnia. Additional research is also needed to establish a benefit for CBT-I with respect to important psychological outcomes including quality of life.

## Competing interests

The authors declare that they have no competing interests.

## Authors’ contributions

MDM participated in the design and performed the systematic review, created the evidence tables, coordinated the writing and editing, and wrote sections of the manuscript. PG resolved disagreements over article inclusion/exclusion, provided clinical context for the systematic review, and wrote sections of the manuscript. MP provided clinical context for the systematic review and wrote sections of the manuscript. CAU participated in the design of the systematic review, reviewed the results, and wrote sections of the manuscript. All authors reviewed, co-edited, and approved the final manuscript.

## Funding

No external funds were used to support this review, and no external companies or organizations were involved in the design or reporting of the review.

## Pre-publication history

The pre-publication history for this paper can be accessed here:

http://www.biomedcentral.com/1471-2296/13/40/prepub

## Supplementary Material

Additional file 1 **Table S1.** OVID Medline search–articles published 1950 through September Week 3 2011. **Table S2.** Cochrane Central Register of Controlled Trials search–2011 issue 3. **Table S3.** EMBASE search–articles published 1974 through September 2011. **Table S4.** PsycINFO search–inception through September 2011. **Table S5.** Excluded studies. **Table S6.** CEP composite rating scale for quality of RCTs. **Table S7.** Study-by-study quality assessment [43-55]. Click here for file
