# Supplementary material for: Comparative effectiveness of cognitive behavioral therapy for insomnia: a systematic review
Source: BMC Fam Pract. 2012 May 25;13:40. doi: 10.1186/1471-2296-13-40 (PMC3481424; doi:10.1186/1471-2296-13-40)
Supplement: Additional file 1 — Table S1. OVID Medline search–articles published 1950 through September Week 3 2011. Table S2. Cochrane Central Register of Controlled Trials search–2011 issue 3. Table S3. EMBASE search–articles published 1974 through September 2011. Table S4. PsycINFO search–inception through September 2011. Table S5. Excluded studies. Table S6. CEP composite rating scale for quality of RCTs. Table S7. Study-by-study quality assessment [43-55]. [file 1471-2296-13-40-S1.doc]

# Supplemental Material

Supplemental table 1 OVID Medline search–articles published 1950 through September Week 3 2011

| **Search** | **Syntax** | **Hits** | **Retrieved** | **Included** |
| --- | --- | --- | --- | --- |
| 1 | exp behavior therapy/ | 44,855 | — | — |
| 2 | insomnia.mp or exp “sleep initiation and maintenance disorders”/ | 12,428 | — | — |
| 3 | 1 and 2 | 779 | — | — |
| 4 | (randomized controlled trial or controlled clinical trial).pt. or randomized controlled trials.sh. or random allocation.sh. or double blind method.sh. or single blind method.sh. | 467,012 | — | — |
| 5 | 3 and 4 | 164 | — | — |
|  | Delete 20 duplicate records within set | 144 | 20 | 5 |

Supplemental table 2. Cochrane Central Register of Controlled Trials search–2011 issue 3

| **Search** | **Syntax** | **Hits** | **Retrieved** | **Included** |
| --- | --- | --- | --- | --- |
| 1 | MeSH descriptor “Sleep Initiation and Maintenance Disorders” explode all trees | 1,044 | — | — |
| 2 | MeSH descriptor “Cognitive Therapy” explode all trees | 3,057 | — | — |
| 3 | #1 and #2 | 71 | — | — |
|  | All references duplicated Medline results | 0 | 0 | 0 |

Supplemental table 3. EMBASE search–articles published 1974 through September 2011

| **Search** | **Syntax** | **Hits** | **Retrieved** | **Included** |
| --- | --- | --- | --- | --- |
| 1 | 'insomnia'/exp/mj | 9,628 | — | — |
| 2 | 'cognitive therapy'/exp/mj | 8,332 | — | — |
| 3 | 1 and 2 | 192 | — | — |
| 4 | 3 and ([controlled clinical trial]/lim OR [randomized controlled trial]/lim) | 50 | — | — |
|  | delete 36 references duplicating Medline results | 14 | 2 | 0 |

exp–explode all trees, mj–restrict to major focus of article

Supplemental table 4. PsycINFO search–inception through September 2011

| **Search** | **Syntax** | **Hits** | **Retrieved** | **Included** |
| --- | --- | --- | --- | --- |
| 1 | mjsub(insomnia) | 2,782 | — | — |
| 2 | cognitive behavioral therapy | 25,131 | — | — |
| 3 | 1 and 2 | 273 | — | — |
| 4 | methodology is ME=(prospective study) or ME=(quantitative study) or ME=(treatment outcome/clinical trial) | 95 | — | — |
|  | title and abstract screen | 10 | — | — |
|  | delete 9 references duplicating previous results | 1 | 1 | 0 |

Supplemental table 5. Excluded studies

| **Study** | **Comparison** | **Reason for exclusion** |
| --- | --- | --- |
| Gross 2011 [43] | Stress reduction vs. eszopiclone | Study intervention was not CBT-I |
| Blumer 2009 [44] | Zolpidem vs. placebo | No CBT-I intervention |
| Morin 2009 [45] | CBT-I plus zolpidem vs. CBT-I alone | Did not measure effectiveness of CBT-I |
| Manber 2008 [46] | CBT-I and escitalopram vs. escitalopram alone | Both study groups given medication |
| Belleville 2007 [47] | Self-help CBT-I with and without med. taper | No provider element of CBT-I |
| Carney 2007 [48] | CBT-I vs. anti-depressant | Cross-sectional design |
| Vallières 2005 [49] | CBT-I vs. zopiclone | Too few patients |
| Perlis 2004 [50] | CBT-I vs. modafinil | Too few patients |
| Vallières 2004 [51] | CBT-I vs. zopiclone | Too few patients |
| Morin 2003 [41] | CBT-I and tanezapam vs. tanezapam alone | Subset of included study [31] |
| Waters 2003 [52] | Cognitive distraction vs. flurazepam | Compared components of CBT-I |
| Thase 2001 [34] | CBT-I vs. nefazodone | Drug withdrawn from US market |
| Pimlott 2000 [53] | No original data | Commentary on published study [31] |
| Hauri 1997 [54] | Relaxation therapy vs. triazolam | Too few patients |
| Milby 1993 [55] | CBT-I vs. triazolam | Too few patients |

Supplemental table 6. CEP composite rating scale for quality of RCTs

Randomization:

1. J Described as randomized?

2. J Randomization appropriately performed?

Blinding:

3. J Study described as double-blinded?

4. C Outcome assessor blinded?

5. J Study participant blinded (e.g. intervention described as indistinguishable, active placebo, identical placebo or dummy)?

6. C Investigator blinded?

Patient attrition:

7. J Attrition described?

8. C Attrition smaller than 10-15% of assigned patients?

9. C Attrition appropriately analyzed (i.e. intention-to-treat analysis for superiority studies)?

Quality rating for GRADE analysis

Rating of evidence base was lowered by one grade if conclusions were dependent on an individual study with quality score of 6 or less, and by two grades if conclusions were dependent on an individual study with quality score of 4 or less.

J–components from original Jadad scale [22]; C–components from Chalmers list [23]

Supplemental table 7. Study-by-study quality assessment

| Study | Described as  randomized? | Randomization  appropriately performed? | Described as  double-blinded? | Outcome assessor blinded? | Study participant  blinded? | Investigator  blinded? | Attrition  described? | Attrition less than 10-15% ? | Attrition appropriately  analyzed? | Total |
| --- | --- | --- | --- | --- | --- | --- | --- | --- | --- | --- |
| CBT-I vs. zopiclone | | | | | | | | | | |
| Sivertsen 2006 [29] | 1 | 0 | 1 | 1 | 0 | 0 | 1 | 0 | 1 | 5 |
| CBT-I vs. zolpidem | | | | | | | | | | |
| Jacobs 2004 [30] | 1 | 1 | 0 | 0 | 0 | 0 | 1 | 1 | 1 | 5 |
| CBT-I vs. temazepam | | | | | | | | | | |
| Wu 2006 [32] | 1 | 0 | 0 | 0 | 0 | 0 | 1 | 1 | 0 | 3 |
| Morin 1999 [31] | 1 | 0 | 1 | 1 | 0 | 0 | 1 | 1 | 1 | 6 |
| CBT-I vs. triazolam | | | | | | | | | | |
| McCluskey 1991 [33] | 1 | 0 | 0 | 0 | 0 | 0 | 1 | 1 | 1 | 4 |

# References (supplemental material)

43. Gross CR, Kreitzer MJ, Reilly-Spong M, Wall M, Winbush NY, Patterson R, Mahowald M, Cramer-Bornemann M: **Mindfulness-based stress reduction versus pharmacotherapy for chronic primary insomnia: a randomized controlled clinical trial.** *Explore: The Journal of Science & Healing* 2011, **7**: 76-87.

44. Blumer JL, Findling RL, Shih WJ, Soubrane C, Reed MD: **Controlled clinical trial of zolpidem for the treatment of insomnia associated with attention-deficit/hyperactivity disorder in children 6 to 17 years of age.** *Pediatrics* 2009, **123**: e770-e776.

45. Morin CM, Vallieres A, Guay B, Ivers H, Savard J, Merette C, Bastien C, Baillargeon L: **Cognitive behavioral therapy, singly and combined with medication, for persistent insomnia: a randomized controlled trial.** *JAMA* 2009, **301**: 2005-2015.

46. Manber R, Edinger JD, Gress JL, San Pedro-Salcedo MG, Kuo TF, Kalista T: **Cognitive behavioral therapy for insomnia enhances depression outcome in patients with comorbid major depressive disorder and insomnia.** *Sleep* 2008, **31**: 489-495.

47. Belleville G, Guay C, Guay B, Morin CM: **Hypnotic taper with or without self-help treatment of insomnia: a randomized clinical trial.** *J Consult Clin Psychol* 2007, **75**: 325-335.

48. Carney CE, Segal ZV, Edinger JD, Krystal AD: **A comparison of rates of residual insomnia symptoms following pharmacotherapy or cognitive-behavioral therapy for major depressive disorder.** *J Clin Psychiatry* 2007, **68**: 254-260.

49. Vallieres A, Morin CM, Guay B: **Sequential combinations of drug and cognitive behavioral therapy for chronic insomnia: an exploratory study.** *Behav Res Ther* 2005, **43**: 1611-1630.

50 Perlis ML, Smith MT, Orff H, Enright T, Nowakowski S, Jungquist C, Plotkin K: **The effects of modafinil and cognitive behavior therapy on sleep continuity in patients with primary insomnia.** *Sleep* 2004, **27**: 715-725.

51. Vallières A, Morin CM, Guay B, Bastien CH, LeBlanc M: **Sequential treatment for chronic insomnia: a pilot study.** *Behav Sleep Med* 2004, **2**: 94-112.

52. Waters WF, Hurry MJ, Binks PG, Carney CE, Lajos LE, Fuller KH, Betz B, Johnson J, Anderson T, Tucci JM: **Behavioral and hypnotic treatments for insomnia subtypes.** *Behav Sleep Med* 2003, **1**: 81-101.

53. Pimlott NJ: **Pharmacologic or behavioural therapy for elderly people's insomnia. Which is better?** *Can Fam Physician* 2000, **46**: 1430-1432.

54. Hauri PJ. **Can we mix behavioral therapy with hypnotics when treating insomniacs?** *Sleep* 1997, **20**: 1111-1118.

55. Milby JB, Williams V, Hall JN, Khuder S, McGill T, Wooten V. **Effectiveness of combined triazolam-behavioral therapy for primary insomnia.** *Am J Psychiatry* 199, **150**: 1259-1260.
